# Considering Patients’ Empowerment in Chronic Care Management: A Cross-Level Approach

**DOI:** 10.3390/ejihpe10010012

**Published:** 2019-10-18

**Authors:** Caroline Tilkin, Mélanie De Winter, Frédéric Ketterer, Anne-Marie Etienne, Marc Vanmeerbeek, Frédéric Schoenaers

**Affiliations:** 1 Health and Society Interfaculty Unit, University of Liège, 4000 Liège, Belgium; am.etienne@uliege.be; 2 Social Sciences Research Institute, University of Liège, 4000 Liège, Belgium; mdewinter@uliege.be (M.D.W.); f.schoenaers@uliege.be (F.S.); 3 Primary Care & Health Research Unit, University of Liège, 4000 Liège, Belgium; frederic.ketterer@yahoo.fr (F.K.); marc.vanmeerbeek@uliege.be (M.V.)

**Keywords:** empowerment, patient, integrated care, cross-level analysis

## Abstract

This paper consists of an analysis of the concept of empowerment—which is often defined as a key issue in health care—at the macro, meso, and micro levels by focusing on health care reform in Belgium. Three research teams collected data and combined them in an inductive secondary analysis. Our preliminary results demonstrate that patient empowerment does not always encompass the same scientific reality. At the macro level, this concept is linked to the authorities’ wish to support at-home care for chronic patients. At the meso level, the role of caregivers in maintaining patients’ autonomy, but also the social conditions of their lives, is a salient component of empowerment. At the micro level, individual and personal features such as identity can influence patient empowerment and behavior in the health care system. This cross-level research suggests that patient empowerment is not sufficiently clearly defined at each level of the care production chain, which could impede the reform of health care. This paper underlines the polysemy of a concept viewed as a milestone in European health care policy and the necessity of a clear, collective definition to operationalize and implement it.

## 1. Introduction

The ongoing increase in chronic illness poses new challenges for European countries. In Belgium, more than one in four people aged 15 and over have at least one chronic condition [1]. According to the literature, this situation has led to an increase in health care expenditures [2,3,4]. In this context, the Belgian Government has decided to initiate a health care reform through the launch of a joint plan called “Integrated Care for Better Health” (IC4BH) [5]. The authorities have chosen to implement this joint plan through an iterative, incremental process, by launching multidisciplinary local pilot projects involving a variety of local (hospitals, medical health centers, general practitioners, ambulatory home care nursing clinics, representatives of patients and informal caregivers, etc.). 

One of the main goals of this plan is to shift from a fragmented system to an integrated and more competitive one. The World Health Organization (WHO) refers to care integration as “the organization and management of health services so that people get the care they need, when they need it, in ways that are user friendly, achieve the desired results, and provide value for money” [6]. Dealing with the problem of chronic diseases necessitates this kind of paradigm change. With this aim in mind, the plan (IC4BH) was structured into 20 central components, including patient empowerment [5]. 

According to Aujoulat et al., the concept of empowerment is rooted in an ideology that emerged in social work during the 1960s. At that time, the focus was on individuals’ and communities’ rights and competences more than their needs and shortfalls [7]. At the end of the 1970s, the term *empowerment* was widely used in domains as varied as social services, public health, community development, etc. [8]. In the specific domain of health, the WHO’s Ottawa Charter for Health Promotion in 1986 made empowerment a cornerstone of health promotion; it promoted a positive definition of health that is not limited to the prevention of health issues, but rather stresses social circumstances and their improvement [7]. In this context, health promotion was seen as “the process of enabling people to increase control over, and to improve, their health” [9].

Nowadays, empowerment is often considered to be an “umbrella term”, meaning that many interpretations of this concept exist. It can be defined simultaneously as a process, an education strategy, or even an outcome [7]. Other terms are sometimes used in place of *empowerment* such as *patient participation* or *patient-centered care*, which can lead to a lack of clarity for researchers, patients, health care providers, and policy makers [10]. However, the crux of this concept is the idea of helping patients to become more responsible for their own health, by giving individuals and community groups more power over the definition and nature of changes affecting them [11]. Indeed, according to many experts, “chronic diseases are managed most effectively when patients take an active role in this themselves” [12]. Empowerment can be considered a health-enhancing process, which led us to raise some questions about the different meanings concealed behind the term *empowerment* in the context of the Belgian care management chain [7,12].

The goal of this study was to investigate with an inductive perspective how the concept of empowerment is viewed at each level of the Belgian health care production chain by the different stakeholders involved or concerned by the reform (political, institutional, caregivers, patients). This multifaceted concept can have a lot of different meanings, as shown above. In addition, in the Belgian reform, patient empowerment is defined as an ongoing process in the course of care of the patient and his or her caregiver [5]. What are the consequences of such an elusive concept when different health care stakeholders have to work together? Our purpose here was to try to highlight this issue in the context of integrated care. Indeed, the scientific literature stresses the need for a greater understanding of the concept of empowerment to guide clinical care, research, and health systems to create powerful interventions and policies [13]. Consequently, we decided to undertake an inductive analysis of empowerment, starting from empirical conceptions expressed by the stakeholders involved in the chronic care reform, at the different levels of the health care production chain. 

## 2. Materials and Methods 

To answer this research question, three research teams, coming from three different schools (social sciences, medicine, and psychology), decided to combine the results of their respective studies to produce a new inductive analysis. Therefore, this article presents a secondary analysis of data that aims to explain how the concept of empowerment is expressed at different levels. Each research team worked on a specific level of the care production chain, identified as the macro, meso, and micro levels. The macro level considers political and organizational aspects. The meso and micro levels take therapeutic aspects into account, from the perspective of health practitioners, informal caregivers, and sick people (meso level) to that of patients (micro level). In the context of health care, we have to consider that these three levels are embedded [14]. Consequently, they are interdependent in our analysis. A more exhaustive presentation of the aim of each study was presented in 2018 at the International Conference on Integrated Care and is summarized in Table 1 [15].

## 3. Results

This study was an initial step in integrating a cross-level perspective on empowerment. Our results highlight the inherent complexity of the concept through the point of view of the various stakeholders involved, the levels of analysis (macro, meso, and micro), and the conditions on the ground. 

### 3.1. Macro Level 

The results of our qualitative analyses show that empowerment is a major component of the Belgian joint plan and entails a major shift in terms of professional practice. Historically, the Belgian health care system developed in response to the challenges posed by acute diseases. Progressively, the system became increasingly specialized and fragmented. As a result, the predominance of chronic diseases led to the emergence of a paradoxical situation: in its current state, the Belgian health care system does not meet chronic patients’ needs. The high degree of specialization and the lack of cooperation between practitioners that characterize the current system impede the delivery of care adapted to chronic patients’ specific characteristics [5]. By definition, those patients are not expected to be cured; instead, they must deal with their condition in the long run (see [12]). In their case, the issue is not to cure them quickly, but rather to help them live with their chronic disease(s) at home over the long term, which raises questions of social integration. 

In IC4BH, patient empowerment is mainly defined as providing support and information for chronic patients, who are expected to play a more active role in their own care, and is also viewed as necessary to reduce hospital stays and costs. This principle is supposed to help create a new type of therapeutic relationship that is more patient-centered. In this therapeutic configuration, patients’ empowerment appears to be important, which explains why it was identified as major component of IC4BH. Chronic patients do need to be able to deal with their conditions and care for themselves on a daily basis, even if they are not in hospital or if no health care professional is there to assist them. 

*“And what happens if the patient can’t be empowered?”* (Home care worker, Field observation notes, 2017). On the basis of their professional experience, many pilot project stakeholders argued that certain patients do not have the ability or the wish to be empowered, and that trying to empower some types of patients can be counterproductive. This shows that, although empowerment is defined as a key issue in the plan, it is often addressed differently by the different pilot project stakeholders, which can lead to misunderstandings. For instance, empowerment does not imply the same concrete professional practice on the ground for a general practitioner (GP) as it does for a health insurance provider. Accordingly, the solutions proposed by each party to foster empowerment generally differ, and will not automatically lead to shorter hospital stays and lower costs. They might even have an opposite effect and increase health care expenditures. 

Behind the concept of empowerment lies the idea of making patients autonomous, of giving them an active role in their own care process so that they can achieve their goals and live as normally as possible, as illustrated in these interview extracts:

*It [the patient’s care trajectory] is not predetermined, because patients who have the same medical status can have different ambitions: staying independent and being able to keep traveling or to look after their kids or … The starting point is what the patient wants … and is able to do, obviously. […] The part about empowerment can also motivate them to seek treatment or look for what could motivate them to seek treatment.*

(High-ranking public official, 2017)

*Forcing the patient to do something which does not correspond to what he/she wants would risk not being effective and, as a result, [would lead to] compliance problems regarding the treatment and unnecessary waste.*

(High-ranking public official, 2017)

For health care professionals, the challenge here is to move from a diseased-centered approach to a patient-centered approach, and consider patients as people whose identity cannot be reduced to their health condition. These elements denote an individual-centered vision of the concept of empowerment, given that they pertain to what patients want as individuals. 

### 3.2. Meso Level 

The triad analysis that was performed among health professionals, sick persons, and family caregivers revealed the limits of the approach taken by the plan for integrated chronic care. First, the sick persons coped in various ways with their health problems and multiple chronic conditions. Some of them had accepted their status as chronically ill people, and had adapted their behavior and lifestyle according to the medical recommendations, while others had not. Since some found it difficult to consider themselves as chronically ill, they were not very invested in the normative expectations concerning them. Their varying acceptance of their status as chronically ill people was linked to the impacts of their diseases on their daily lives: in general, the smaller the practical impact, the less concerned the sick person was [16]. This first observation demonstrates a potential limitation of empowerment: patients who do not recognize their chronically ill status appear to be less likely to modify their behavior and life habits in accordance with medical guidelines or family recommendations. This idea is illustrated in the following two quotations:

*Even after suffering from his embolism and myocardial infarction, there was no awareness of its severity or … Eating was his only… his only pleasure. Therefore, psychologically speaking … even involving psychologists … other providers… Well, he’s not a manageable person.*

(GP about his 60-year-old patient)

*I’ll take my coffee, a cup of coffee; I won’t drink it without sugar. If I have to add sweetener, I find it doesn’t taste the same. Well, in the evening, I’ll take the sweetener if my children are there. That’s really a small detail, isn’t it?*

(70-year-old woman with diabetes and hypertension)

The differences in the subjective appropriation of chronically ill status can also be seen in the way these people coped with the health system. Some patients adapted their behavior and the information they delivered to the health professionals depending on their own interest in and acceptance of their status. This was generally ignored by the health professionals, as well as by some family caregivers, especially if the latter were not living in the same household as the sick persons. This raises the question of which strategy should be prioritized in empowerment: not being reduced to the status of a chronically ill person, as some patients wanted, and thus engaging in behaviors with other objectives than health management, or empowerment in accordance with medical requirements, which may be inconsistent with the sick person’s own objectives.

The following two examples show the possible gap between medical requirements and how patients view their health:

*I agree to make all the efforts required, but … but not to sit all day long while waiting for the day to end. To be allowed to do something, to have little pleasures and eat this or do that and … Except for walking, she (the diabetologist) doesn’t allow me to do anything, you see… […] It’s too much, you know. No, I can’t!*

(70-year-old woman with diabetes and hypertension)

*At this time, we had Glucophage [metformin]. Because I was a sales representative, I was annoyed. It gave me gas, and as I was frequently … I stopped taking this drug. So, each time there was a blood test, they said: “But did you take it …?” “Yes, yes, no problem!” So they increased the amount to take. And I didn’t take it.*

(60-year-old man with diabetes)

In addition to patients’ willingness or unwillingness to comply with their chronically ill status, some external factors may impede the empowerment promoted in the plan for chronic care. The social environment and issues related to people’s life experiences need to be considered. The lives of some of the sick persons interviewed had been marked by serious psychosocial events: financial difficulties, social isolation, or a history of psychiatric disorders. These factors were well known to the GPs, who mentioned them as additional obstacles for chronic condition management. 

The sick people’s presumed intellectual or understanding level was also mentioned as a possible barrier, which could lead GPs to reduce their patients’ accountability, for example for self-measurement device use (blood pressure, blood glucose, etc.).

*Obviously, there are some people I will never give this kind of tool to! Because… well, because they don’t have the brains to cope with it. Clearly. It is … it requires a little judgment to understand and think about.*

(50-year-old GP)

These health professionals’ strategies based on the patients’ social environment reflected the (supposed) reduced relevance of empowerment for some patients.

Finally, empowerment also came up against the working habits and regulatory framework characterizing medical practice. All of the family caregivers interviewed reported inadequate consideration by some health professionals. They particularly singled out hospital specialists, who provided scant information or paid little attention to the impact of diseases on sick people’s daily lives—an aspect that was mainly managed by the family caregivers. The lack of consideration was all the more problematic, given that family caregivers were frequently essential for the sick persons’ home care. The patient-centered approach promoted by health professionals, which they considered as the most relevant, could reduce empowerment to its individual dimension, neglecting the role of family caregivers. Other situations demonstrated that family caregivers exerted social control on sick persons’ behavior, as an extension of the health professionals’ role. The caregivers acted as regulators, ensuring that patients complied with the medical requirements for their disease. Again, empowerment was caught between respecting sick people’s wishes (even if their health management was inappropriate) and adapting their behavior, as required by health professionals and echoed by the family caregivers who supported patients in their daily life activities.

### 3.3. Micro Level 

An exploratory qualitative workshop was conducted with five chronic patients who were cancer survivors: four women and one man with different types of cancer (mean age = 44.2 years). The participants were recruited through a former patients’ association. The content of the workshop was analyzed thematically with the Montreal model in mind [17]. It highlighted the fact that various different self-representations can emerge from former patients after an illness. Since identity after illness can be related to empowerment and patient participation, we were interested to see how chronic patients *identified* characteristics related to their specific identity after illness, *defined* those characteristics, and *articulated* them in the context of their health. The procedure for this workshop with chronic patients and the results are presented below. 

The first step was to consider the terms that chronic patients used to define their illness experiences and their current self-perceptions. These terms and the related detailed features were determined in association with patients’ personal knowledge of their illness (mostly cancer) and the health care system. Workshop participants compiled these features into major categories: global themes emerged and were formed thanks to this first step. As a first result, these categories were broadly related to a new time frame (e.g., illness as an endless process), a new sense of belonging and community (e.g., cancer as a specific experience), and new individual, social, and medical representations (e.g., weakness, stigma, data). Some of the themes were more important for some patients and less for others, but all patients were able to agree on a final selection of themes and features.

*We have to be flexible. While we have already gone through the disease, we have already experienced the disease in our body, then we have to get used to this new “me”, to this new body, to all these limits, but at the same time, we have to adapt to people’s new behaviors, to how people perceive us, to those changing attitudes, and all those changes around us… […] And I would say that adapting to oneself is not the most complicated thing; it’s mostly adapting to others.*

(48-year-old woman, breast cancer survivor)

As a second result, we found that self-representations were not always relevant to understanding empowerment for these patients. Indeed, patient empowerment was never a central category when chronic patients had to define themselves after illness. Thus, each main theme was related to terms or ideas that referred to patient empowerment, engagement, or participation. Patient empowerment was not the door to a new definition of the self; instead, it appeared in subtler ways in terms of patient advocacy, resiliency, changes, and turning points.

A third finding was that patient empowerment emerged differently depending on the life domain (personal, social, or medical). One patient emphasized that the medical discourse did not always allow him to play an active role in his follow-up. However, he could embody this feeling of being actively engaged with his health in his personal and social life, as was shown in some of the self-perceptions he described. To illustrate this observation, we present some extracts from our interviews:

*I had the feeling of being considered as a medical statistic.*

*I don’t want to be seen as a victim by other people, or someone who always complains about his situation. I can take care of myself.*

(59-year-old man, head and neck cancer survivor)

This extract also reveals the patient’s expectations about how he wants to be seen by significant others and by medical staff. More specifically, medical representations were associated with the style of communication used by physicians, a lack of information, and how patients or survivors are considered. Social perceptions were defined in a similar way to medical perceptions and tended to imply risk, weakness, complaints, loneliness, and inconvenience. All these representations contrasted with the way patients defined themselves: *“meaningful”*, *“resilient”*, *“coping”*, *“desire to live”*. These observations demonstrated that the way that identity is impacted after illness is very subjective, and varies depending on the domain. Patient empowerment or participation can be a way to explain these changes, but sometimes guilt and vulnerability prevail in how former patients see themselves. 

Finally, all these elements helped us to formulate new questions about self-representations after illness and how the experience of illness can empower a patient’s identity. However, this study also emphasizes the central ambivalence of the process, which is an ambivalence that was shored up by social and health care perceptions. According to chronic patients, the experience of illness can develop resiliency and empowerment. It can also lead to a “new kind of living”, but it is still seen as a weakness by other people and especially by health practitioners, which does not necessarily lead to patient empowerment.

## 4. Discussion

The aim of this article was to compare—by following an inductive approach—different levels of analysis (macro, meso, and micro levels) and different points of view on patient empowerment in the context of the Belgian health care system. We wanted to understand how the same concept was applied at each level, compare our results with this perspective in mind, and finally see what would emerge from this comparison. We have summarized all our results in Table 2 in order to provide a quick overview of the work, which will be discussed below. 

Regarding meanings, we have already indicated that multiple definitions of empowerment exist, and that the concept is generally used as an “umbrella term” in the scientific literature and in operational documents, as it is also the case in the joint plan defining the Belgian health care reform. Our results reflect the complexity of the concept and show that patient empowerment encompasses a wide range of meanings. A recent literature review has clearly summarized this complexity [18]:

*Patient empowerment is a very complex and paradoxical concept: it is situated at several levels (macro, meso, micro), can be approached on several perspectives (the patient, the health care provider, or the health care system) that lead to different interpretations (a theory, a process, an intervention, an outcome, a feeling, or a paradigm) and surfaces in several areas (e.g., (mental) health and welfare) and disciplines (psychology, sociology, nursing, social work). Different definitions, each with a different emphasis, are consequently in use.*

(p. 1925)

More specifically, at the macro level, the meaning of patient empowerment was determined by IC4BH (2015). This definition tends to focus on different aspects of patient empowerment, which is understood as an individual issue. Therefore, this plan also offers meanings at the micro level, but fails to provide a meso point of view. 

A trend to reduce empowerment to the single dimension of individual capacity is reported in the literature regarding changes in the concept as used in public policies. According to Calvès, although, in its initial acceptance, empowerment was a complex, multidimensional concept that placed the emphasis on the individual and collective dimensions of power, the use of the term in the discourse on development was accompanied by an individualization of the notion of power, which was regarded as individual and economic choices [8]. According to the most judgmental view, “liberator empowerment” has become “liberal empowerment”, which is more focused on maximizing individual interests [19,20]. 

As for expectations related to patient empowerment, we also found contradictory points of view between each level. At the macro level, the economic argument was central in the debate between stakeholders. As a major component of the joint plan, the implementation of empowerment should lead to reduced health care costs and hospital stays. At the meso level, analyzing interactions in triads of physicians, caregivers, and patients reveals that empowerment did not correspond to the authorities’ expectations or to the expectations of patients and their caregivers, who view empowerment according to their own interests and strategies. As an individual issue, patient empowerment does not take all parties into account. At the micro level, expectations concerning patient empowerment should lead to patients’ participation and engagement. However, role reorganization did not always allow this change in patient involvement. 

Finally, regarding real conditions on the ground, there is a gap between the normative definition of empowerment and the way that this concept is understood by actors in the field. They can either use political, professional, or experiential definition depending on the area of expertise to which they belong. The assumption that a scientific definition could be implemented in a similar way in various health fields could not be demonstrated by our analysis on the ground.

## 5. Conclusions

The contrast between meanings, and then expectations, and the implementation of patient empowerment in the field is striking. The polysemy of this concept has made the implementation of patient empowerment more complex. However, the observations collected with our multiple methodologies did not reveal any contradictions with the term “patient empowerment” defined in IC4BH. 

This initial definition of empowerment is connected with the idea that each patient can take responsibility for his/her life and health. However, more specifically, our results showed that empowered patients can decide what they want for their life, which also means that they can decide whether to be treated or not, whether to be hospitalized or not, and whether to be active or not [21]. Consequently, being empowered does not necessarily imply making a decision about health that is related to the guidelines imposed by a plan or by health practitioners. This vision is also coherent with the observation that some patients cannot appropriate an identity that will lead to empowerment or resiliency in the context of their illness. 

Applying a global concept of patient empowerment does not make sense when working at different levels. Moreover, the advantage of the concept of empowerment is that it can have different uses depending on the type of stakeholder. Clear definitions of these terms are essential in order to make them effective and relevant; without them, there’s a risk that empowerment will remain a vague and empty objective that cannot be implemented in the field [22,23].

## Figures and Tables

**Table 1:** Study designs of the macro, meso, and micro levels.

School and Level	Research Question/Aim of the Study	Perspective on Empowerment	Data Collection
**Social Sciences**	Focus on political and organizational governance through the prism of managerial innovation to grasp the whys and wherefores of the ongoing paradigm shift intended to implement integrated care in Belgium.	1. Definition in the plan	Thematic literature review Operational document analysis Qualitative interviews with policy advisors and public officials involved in devising and implementing the new policy, pilot project coordinators, and pilot project stakeholders Direct field observations Focus groups
**Macro Level**	Analysis of the reform design process and the national and international context in which it took place; the rationale for reform; and the specific way the reform was implemented through bottom–up pilot projects.	2. Vision circulated by the authorities	
**Medicine** **Meso Level**	To better grasp the different implications of situations related to chronic illness as experienced by patients and their family caregivers; in particular, acceptance of the social status of chronically ill people, which conditions the place and role of family caregivers, and the manner in which informal and professional caregivers work together.	Perspective of multimorbid patients in relation to professionals and informal caregivers	Qualitative interviews with “triads” (one patient, one of his/her health professionals, and one of his/her informal caregivers) 23 interviews were conducted and analyzed
**Psychology** **Micro Level**	To better understand how having a chronic illness changes chronic patients’ self-representation and to study the risks and protective factors that impact self-representation in patients with different chronic conditions such as cancer, multiple sclerosis, chronic obstructive pulmonary disease, and diabetes.	Perspective of patients stemming from their personal experiences related to their health	Mixed-method study design with qualitative exploratory interviews and scoping research (questionnaires).

**Table 2:** Overview of results.

	Meaning	Expectations	Reality
**Macro Level**	Definition of the joint plan	Shorter hospital stays Cost reductions	Misunderstandings between stakeholders
**Meso Level**	Patient-centered care	Support autonomy Maintain social situation	Collective meaning vs. individual meaning
**Micro Level**	Patient participation: being active and sharing decision making	Foster patients’ involvement Hear patients’ voices and rights	Disruptions in terms of representations and roles
